# Dispersion of Graphite Nanoplates in Polypropylene by Melt Mixing: The Effects of Hydrodynamic Stresses and Residence Time

**DOI:** 10.3390/polym13010102

**Published:** 2020-12-29

**Authors:** Luís Lima Ferrás, Célio Fernandes, Denis Semyonov, João Miguel Nóbrega, José António Covas

**Affiliations:** 1 Centre of Mathematics, Department of Mathematics, University of Minho, 4800-058 Guimarães, Portugal; luislimafr@gmail.com; 2 Department of Polymer Engineering, Institute for Polymers and Composites, Campus de Azurém, University of Minho, 4800-058 Guimarães, Portugal; cbpf@dep.uminho.pt (C.F.); mnobrega@dep.uminho.pt (J.M.N.); 3 Coolbrook, Pieni Roobertinkatu 9, 00130 Helsinki, Finland; denis.semyonov@gmail.com

**Keywords:** polymer nanocomposites, nanofiller dispersion, flow-cells, experimental and numerical, viscoelastic fluids, computational fluid dynamics

## Abstract

This work combines experimental and numerical (computational fluid dynamics) data to better understand the kinetics of the dispersion of graphite nanoplates in a polypropylene melt, using a mixing device that consists of a series of stacked rings with an equal outer diameter and alternating larger and smaller inner diameters, thereby creating a series of converging/diverging flows. Numerical simulation of the flow assuming both inelastic and viscoelastic responses predicted the velocity, streamlines, flow type and shear and normal stress fields for the mixer. Experimental and computed data were combined to determine the trade-off between the local degree of dispersion of the PP/GnP nanocomposite, measured as area ratio, and the absolute average value of the hydrodynamic stresses multiplied by the local cumulative residence time. A strong quasi-linear relationship between the evolution of dispersion measured experimentally and the computational data was obtained. Theory was used to interpret experimental data, and the results obtained confirmed the hypotheses previously put forward by various authors that the dispersion of solid agglomerates requires not only sufficiently high hydrodynamic stresses, but also that these act during sufficient time. Based on these considerations, it was estimated that the cohesive strength of the GnP agglomerates is in the range of 5–50 kPa.

## 1. Introduction

Polymer nanocomposites consisting of a polymer matrix and a small amount of nanofiller, such as layered organoclays, carbon nanotubes, graphene derivatives or their mutual combinations, can present outstanding properties and functionalities, especially when compared with equivalent composites containing micro-sized fillers [1]. The performances of these materials are determined by the inherent properties of their constituents, composition, size, the aspect ratio and specific surface area of the filler, interfacial compatibility and morphology. Currently, it is widely accepted that the optimal performance of polymer nanocomposites can only be fully attained when extensive dispersion of the filler in the matrix is reached. However, most nanoparticles with large surface-to-volume ratios tend to form cohesive macroscopic agglomerates, often bound by van der Waals forces, which hamper dispersion. This difficulty has delayed the wider practical utilization of carbon nanotubes and graphene as fillers for thermoplastic matrices, despite their potential reinforcing effect [2].

Polymer nanocomposites can be manufactured by various routes. In situ polymerization of a low-viscosity monomer in the presence of the filler and of an initiator facilitates wetting, diffusion and infiltration into the agglomerates, yielding good dispersion. In the case of solution processing, filler, polymer and surfactant are mixed with a solvent to lower the viscosity of the medium, followed by evaporation of the solvent. The physical/mechanical mixing of the filler with the polymer in the melt state is known as melt mixing. This is the most attractive method in terms of industrial production, as it avoids the use of solvents, can use well-known polymer processing equipment (often, co-rotating, intermeshing, self-wiping twin screw extruders), is capable of continuous, automatic operation and can achieve high outputs. Nevertheless, the extent of dispersion obtained with this process is usually lesser than that attained when using the other techniques [3]—the resulting nanocomposites contain filler aggregates (smaller that the initial agglomerates), together with individual particles. Thus, in melt mixing, the final degree of dispersion depends on the properties of the ingredients, on the equipment type and geometry and on the operating conditions selected.

Abundant research has been carried out with the aim of establishing correlations between polymer/filler characteristics, mixing conditions, level of filler dispersion and performance of the composite. Studies aiming at understanding the dispersion mechanisms associated with the type of filler and flow conditions are much scarcer. Rwei et al. [4] studied the breakup mechanisms of carbon black agglomerates when subjected to a simple shear flow field. They observed two dispersion modes. Rupture involved rapid break-up of the initial agglomerates into smaller aggregates or into individual particles. Erosion was a much slower process consisting of the progressive detachment of individual particles, or of small aggregates, from the agglomerates. Moreover, the onset of rupture required higher stresses than erosion. This dispersion mechanism was also proposed for carbon nanotubes [5,6]. Rwei et al. [7] investigated the erosion of carbon black agglomerates suspended in Newtonian fluids and found that the kinetics of the process obeys a first order rate equation, that the size of the eroded fragments follows a normal distribution and that the strength of the flow field does affect the kinetics of the dispersion process. The same team developed a model for the dispersion of particle agglomerate suspensions. First, they focused on rupture as the step that primarily determines the dynamics of mixing [8]. The agglomerates were assumed as being clusters of aggregates bound by van der Waals forces. It was concluded that in order to accomplish rupture in simple shear flows, periodic randomization of orientation would be advantageous, but biaxial extension flow fields would be the most efficient flow type for rupture. Later, a model for the erosion kinetics of particle agglomerates in simple shear flows was put forward by Scurati et al. [9]. The study included dispersion experiments using silica agglomerates of various densities and polydimethyl(siloxane) of different viscosities. The overall model defined a fragmentation number (*Fa*) that balances the hydrodynamic stresses against the cohesive strength of the agglomerates. For the silica system studied, agglomerates eroded for 2≤Fa<5, while rupture became predominant for Fa>5. Even for high values of *Fa*, there was a finite probability associated with break-up that is proportional to the residence time and agglomerate surface area. A comprehensive review on solid agglomerate dispersion can be found in [10].

The effectiveness of extensional flows for dispersive mixing was demonstrated experimentally by Grace for Newtonian fluid–fluid systems [11]. When the viscosity ratio becomes greater than four, simple shear flows cannot resolve the interfacial tension and suspended droplets are not broken up. Contrarily, critical capillary numbers are lower for elongational flows. No limit exists for the viscosity ratio. As discussed by Astarita [12] and Tokihisa et al. [13], while shear flows induce rotational motion as a result of vorticity, and the changes in conformation and orientation are generally limited, extensional flows create stronger flows without vorticity and therefore should be significantly more effective in stretching and orienting chains.

**Figure 1:**
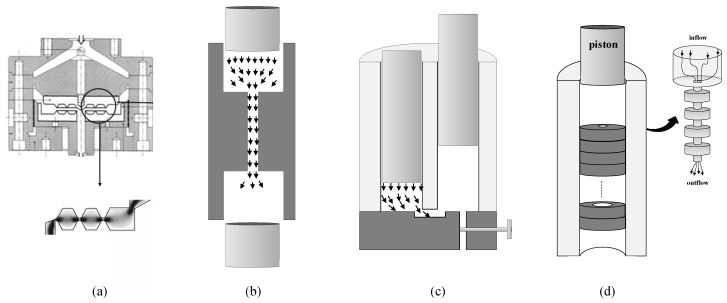
Schematics of extensional mixers: (**a**) Extensional Flow Mixer; (**b**) RMX^®^mixer; (**c**) Son’s [18] internal mixer; (**d**) mixer used in this work.

Based on these concepts, various mixing devices have been developed aiming at creating a strong extensional flow component in addition to the shear flow. They are either attachments to be used in conventional processing equipment, or were conceived as standalone apparatuses. The Extensional Flow Mixer consists of a series of annular convergent-divergent channels located between the extruder and the shaping die (Figure 1a) [14]. In each convergence, the flow is subjected mostly to shear stresses at the walls and primarily extensional stresses along the centerline, due to the acceleration of the flow at the centerline relative to the wall. Carson et al. [15] designed a stationary extensional mixing element for co-rotating twin-screw extruders, containing hyperbolically contracting channels. The RMX^®^mixer [16] has similarities with the multipass rheometer [17]. Two opposite cylindrical chambers are separated by a removable die. Two reciprocally moving hydraulic pistons generate convergent and divergent elongational flows at the entrance and exit of the die, respectively (Figure 1b). In the design proposed by Son [18] the two cylindrical chambers (containing reciprocal cylindrical pistons) are parallel and connected through a narrow rectangular channel (Figure 1c). A modified capillary rheometer set-up, containing a series of stacked rings with an equal outer diameter but alternating larger and smaller inner diameters, was also utilized [19]. The material is forced down through the rings by the piston of the capillary rheometer, being subjected to a sequence of convergent-divergent flows (Figure 1d). Once an experiment is completed, the device can be quickly removed from the rheometer, and opened to collect samples for subsequent morphological characterization. In order to study both dispersion and relaxation phenomena, the design was later modified by inserting a relaxation chamber (where the melt is subjected to quasi-quiescent conditions) between two series of stacked rings with alternating larger and smaller inner diameters [20].

Numerical methods can access pressure, velocity, temperature and stress profiles in a given flow channel. Coupling the flow description to dispersion and/or distribution models yields the capacity to predict the influences of material characteristics, equipment geometry and operating conditions, on the kinetics of morphology development. For example, Bourry et al. [14] used the boundary element method to model flow and drop deformation and breakup through the Extensional Flow Mixer. A model for solid agglomerate dispersion in single-screw extruders was developed by Domingues et al. [21], combining numerical simulations of flow patterns in the metering section of the screw with a Monte Carlo method of cluster rupture and erosion assessed by a local fragmentation number. The model was applied successfully to predict the dispersion of micronized silica in a high-density polyethylene melt [22]. Berzin et al. [23] proposed a model of agglomeration/breakup of inorganic fillers to predict dispersion in a polymer matrix along a co-rotating twin-screw extruder as a function of the screw geometry and processing conditions. Bumm et al. [24] investigated experimentally and numerically the break-up of silica agglomerates in a co-rotating twin-screw extruder. Connelly and Kokini [25] used a 2D finite element method and particle tracking to predict distributive and dispersive mixing in single screw and co-rotating twin screw dough mixers. Valette et al. [26] developed a general finite element code devoted to the 3D simulation of mixing processes involving the flow of generalized Newtonian fluids, which was applied to the simulation of the dispersion of solid particles in an internal mixer, by coupling to a theory of dispersion kinetics.

This work combines experimental and numerical data to better understand the evolution of dispersion of graphite nanoplates in the mixing device consisting of a series of stacked rings with an equal outer diameter and alternating larger and smaller inner diameters (Figure 1d). This mixer was used to manufacture nanocomposites containing carbon nanofibers, carbon nanotubes or graphite nanoplates, yielding levels of dispersion comparable to those typically attained with twin-screw extruders [19,20,27]. The numerical simulations considered both inelastic and viscoelastic fluids. The polymer melt was rheologically characterized, and a six-mode Giesekus model was used to fit the rheological data. This work deals with the mixing of graphite nanoplates in polymer melts. For pre-impregnated composite materials, please se the work by Teodorescu-Draghicescu and Vlase [28].

The remainder of this paper is organized as follows: First, the governing equations for both generalized Newtonian and viscoelastic non-Newtonian fluids, and the numerical method, are presented. Then, the experimental set-up is described, the experimental data obtained are shown and the polymer melt is characterized rheologically. Flow through the mixing device considering 4:1 and 8:1 contractions and expansions is discussed. Finally, experimental and computed data are combined, analyzed and discussed.

## 2. Governing Equations

The equations governing the confined flow of incompressible fluids are the continuity equation

(1) ∇·u=0,

and the momentum equation

(2) $$\rho \frac{\partial u}{\partial t}+\rho \nabla \cdot \left(uu\right)=-\nabla p+\nabla \cdot \tau ,$$

where u is the velocity vector, *p* is the pressure, ρ is the density and $\tau =\tau_{s}+\tau_{p}$ is the deviatoric stress tensor. The stress tensor is divided into a solvent contribution, $\tau_{s}=2\eta_{s}D$ (with $\eta_{s}$ being the solvent viscosity and $D=\frac{1}{2}\left(\nabla u+{\left(\nabla u\right)}^{T}\right)$ the rate of deformation tensor) and a polymer contribution, $\tau_{p}=\sum_{i}^{n}\tau_{pi}$ which in this case is given by the Giesekus [29] *n*-mode model (a constitutive model based on the concept of configuration-dependent molecular mobility):

(3) $$\tau_{pi}+\lambda_{i}\left(\frac{\partial \tau_{pi}}{\partial t}+u\cdot \nabla \tau_{p}-\left[{\left(\nabla u\right)}^{T}\cdot \tau_{pi}+\tau_{pi}\cdot \nabla u\right]\right)+\frac{\alpha_{i}\lambda_{i}}{\eta_{pi}}\left(\tau_{pi}\cdot \tau_{pi}\right)=\eta_{pi}\left(\nabla u+{\left(\nabla u\right)}^{T}\right)$$

where $\lambda_{i}$ is the relaxation time and $\eta_{pi}$ is the polymer viscosity coefficient. The nonlinear terms in the stress that are present in the Giesekus model result from including anisotropy in the hydrodynamic drag and the Brownian motion forces on Hookean polymer molecules—α being the mobility parameter associated with such anisotropy. Note that α controls the extensional viscosity and the ratio of the second normal stress compared to the first one. For α=0 and $\eta_{s}=0$ the model becomes the isotropic UCM model, and for α=1 anisotropic drag is reached. When α>0 we obtain a shear thinning viscosity. The Giesekus model predicts the stress-thickening region for elongational flow, after which a plateau is reached and a stress-thinning region develops at high strain rates. The Giesekus model is able to describe shear-thinning, $N_{1}$ and $N_{2}$, nonlinear time effects, finite extensional viscosity, non-exponential stress relaxation and stress-overshoots using a single nonlinear parameter.

For inelastic fluids $\tau_{p}=0$. Based on invariance requirements, thermodynamic considerations and assuming the behavior of real fluids, $\eta_{s}$ is a function of the second invariant of the rate of the deformation tensor $\left(II_{D}=-\frac{1}{2}tr\left(D^{2}\right)\right)$. Let $S=-4II_{D}$ (so that a positive quantity is obtained, and for simple shear flows $S={\dot{\gamma }}^{2}$); then, the Bird–Carreau generalized Newtonian model, which is the inelastic model adopted in this work, can be written as

(4) $$\eta_{s}\left(S\right)\equiv \eta \left(S\right)=\eta_{\infty }+\frac{\eta_{0}-\eta_{\infty }}{{\left(1+\lambda^{2}S\right)}^{\frac{1-n}{2}}}$$

where $\eta_{0}$ and $\eta_{\infty }$ are the zero and infinite shear rate viscosities, respectively, and *n* is a dimensionless parameter.

In order to evaluate the type of flow distribution, we use the flow type parameter, ξ, defined as:

(5) $$\xi =\frac{\left|D\right|-\left|\Omega \right|}{\left|D\right|+\left|\Omega \right|}$$

where $\left|D\right|$ and $\left|\Omega \right|$ represent the magnitudes of the rate of deformation (D) and vorticity ($\Omega =1/2[\nabla u-{\left(\nabla u\right)}^{T}]$) tensors, respectively. These are given by,

(6) $$\begin{array}{ccc} \left|D\right| & = & \sqrt{\frac{1}{2}\left(D:D^{T}\right)}=\sqrt{\frac{1}{2}\sum_{i}\sum_{j}D_{ij}^{2}}\\ \left|\Omega \right| & = & \sqrt{\frac{1}{2}\left(\Omega :\Omega^{T}\right)=}\sqrt{\frac{1}{2}\sum_{i}\sum_{j}\Omega_{ij}^{2}}. \end{array}$$

The flow type parameter varies from −1, which corresponds to solid-like rotation, up to 1, for pure extensional flow. Pure shear flow is characterized by ξ=0.

This measure of flow type was originally introduced by Giesekus [30] and later used by Fuller and Leal for homogeneous flows [31]. For more on this subject, see [32].

## 3. Numerical Method and Meshes

The systems of Equations (1)–(3) (for viscoelastic fluids) and (1), (2) and (4) (for inelastic fluids) were solved using a methodology based on the finite volume method and the opensource OpenFOAM software [33].

The PISO (pressure implicit with splitting of operators) method was used to couple velocity, pressure and (for the case of viscoelastic fluids) stress fields [34]. The linear systems of equations resulting from the discretisation of the momentum and constitutive equations were solved with the biconjugate gradient stabilized method; for the pressure, the conjugate gradient method was used together with a GAMG preconditioner. The discretization schemes and methods used are: central differences for the diffusive terms, Van-Leer [35] for the convective terms and Euler method for the time derivative. Note that the evolution in time was used just for relaxation purposes.

**Figure 2:**
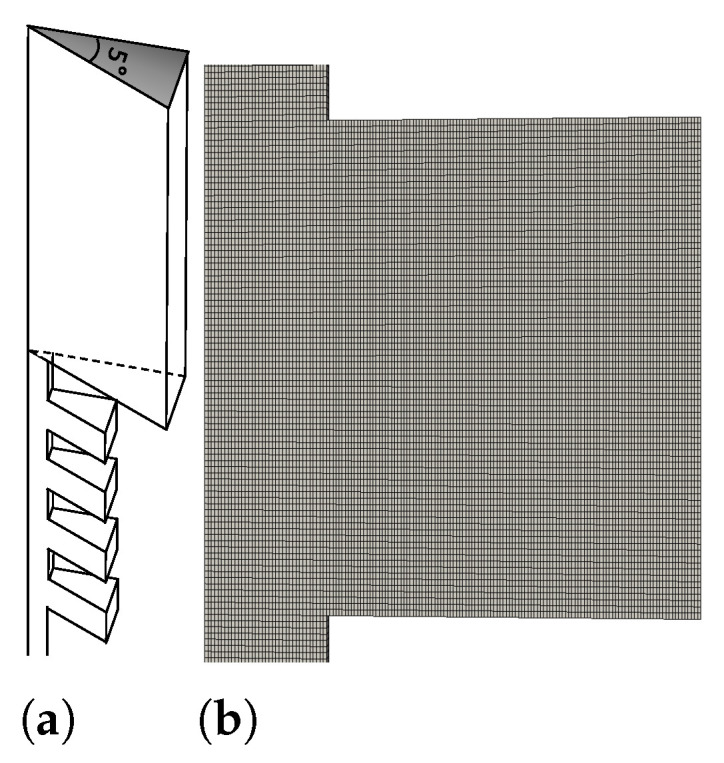
(**a**) Melt mixer geometry: Series of rings with alternating diameters in a capillary rheometer. Due to symmetry, only a wedge of the geometry was considered. (**b**) Zoomed-in view of the mesh used in the simulations.

The simulations were performed using three different progressively refined meshes taking into account feasible computational times. This enabled better control of the convergence and accuracy of the results. The simulations were performed in the confined geometry shown in Figure 2a, i.e., only a slice of the geometry was considered, assuming axisymmetric flow conditions. In order to perform axisymmetric simulations with OpenFOAM, a wedge boundary condition was used, together with the suggested five degree angle for the slice, as shown in Figure 2a.

Part of the intermediate mesh used in the simulations is shown in Figure 2b. The number of cells was 735,384. Capturing the precise vortex dimensions would require a more refined mesh, but that was not the objective of this work. This mesh refinement proved to be sufficient, as it allowed capturing all the relevant flow features.

## 4. Experimental Dispersion Data and Rhelogical Characterization

### 4.1. Dispersion Experiments

The dispersion experiments were performed using the following materials [20]:

- As matrix, a polypropylene copolymer (PP) (Icorene CO14RM from Ico Polymers, France, with a melt flow index of 13.0 g/10 min @190 ${}^{\circ }$C/ 2.16 kg and a density of 0.9 g/cm${}^{3}$);
- As filler, graphite nanoplates (GnP) (Grade C-750 from XG Sciences, Inc., Lansing, MI, USA, with a size distribution ranging from very small (100 nm) to relatively large flakes (1–2 μm)).

The prototype mixer was attached to a Rosand RH8 capillary rheometer and comprised a reservoir, where the polymer and GnP (98/2 wt./wt.%) were fed in powder form and heated to a melt, followed by a vertical stack of ten 2 mm thick circular rings with alternating internal diameters (1 and 8 mm). This set-up created a series of five converging/diverging (8:1 and 1:8) channels. The assemblage of rings was mounted inside a sleeve that can be quickly removed from the body of the device.

After pre-heating for 5 min to 200 ${}^{\circ }$C, the piston of the rheometer moves downwards at constant speed, forcing the melt through the mixer and out of the device as a continuous filament. Three piston speeds of 15, 50 and 100 mm/min were tested, corresponding to average shear rates of approximately 450 $s^{-1}$, 1500 $s^{-1}$ and 3000 $s^{-1}$, respectively. Once the experiment was finished, the sleeve containing the rings was quickly removed and the individual rings were detached from each other. The materials contained in the 8 mm rings were collected and immediately immersed in liquid nitrogen, to freeze the GnP dispersion morphology. In this way, samples of the nanocomposite along the axis of the mixer were made available. The dispersion of the GnP agglomerates was assessed by transmission optical microscopy. Nanocomposite sections 5 μm thick were obtained using a Leitz 1401 microtome, Wetzlar, Germany equipped with glass knives with an angle of 45${}^{\circ }$ and operating at room temperature. Micrographs were acquired with a Leica DFC 280 digital camera coupled to a BH2 Olympus microscope, Tokyo, Japan (with a 20× objective and 1.6× ocular magnification). Quantitative particle analysis was carried out using the Leica Application Suite 4.4 software, Wetzlar, Germany, for at least 5 different images for each sample.

Quantitative characterization of dispersion is complex, since the results are influenced by various parameters, such as nanoparticle size, shape and orientation. Different authors have adopted distinct dispersion assessment strategies; the matter is still open to debate. The use of a global index, even if eventually less precise, facilitates comparison and ranking of different samples. One such popular index is area ratio ($A_{r}$), which is defined as the fraction of composite area occupied by agglomerates, calculated as the ratio of the sum of the areas of all agglomerates measured, to the total composite area analyzed.

**Figure 3:**
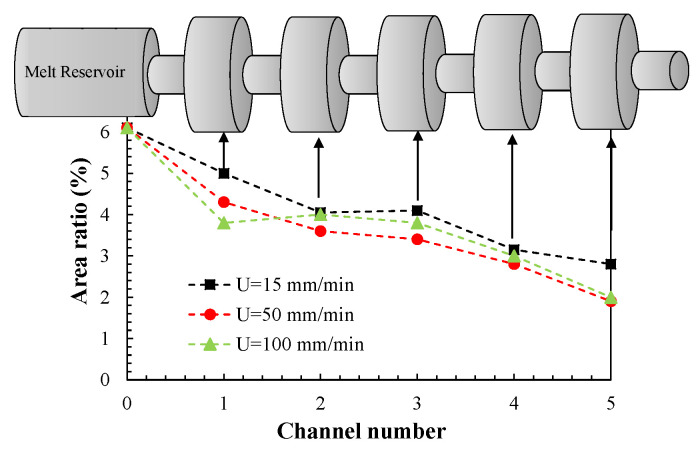
Variation of the area ratio along the mixer: experimental results.

Figure 3 shows the evolution of GnP agglomerate dispersion (in terms of $A_{r}$) along the mixer, for the three piston speeds utilized. Within the experimental error, it is difficult to perceive a clear effect of piston speed. Probably, the non-Newtonian character of the flow reduces the potential effects of variations in shear and extensional rate. The data show that the threshold stress for rupture or erosion of the agglomerates is attained even at the lowest piston speed. The evolution of $A_{r}$ along the mixer is basically linear, the rate probably depending on the intensity of the hydrodynamic stresses. This gradual dispersion along the mixer is quite different from that observed for PP/carbon nanotube (CNT) nanocomposites prepared in the same device, where a stepwise evolution was seen [19,27]. This behavior was taken as evidence of the rupture of CNT agglomerates when exposed to a certain stress level during a given time; erosion developed in between-steps, as hypothesized by Scurati et al. [9]. In the present case, it is difficult to distinguish whether erosion, rupture or a combination of both prevailed.

### 4.2. Rheological Characterization

The PP matrix was characterized at three temperatures (180, 200 and 220 ${}^{\circ }$C) using a stress controlled rotational rheometer (Paar Physica MCR 300, Graz, Belgium) and a capillary rheometer (Rosand RH10, NETZSCH-Gerätebau GmbH, Selb, Germany).

**Figure 4:**
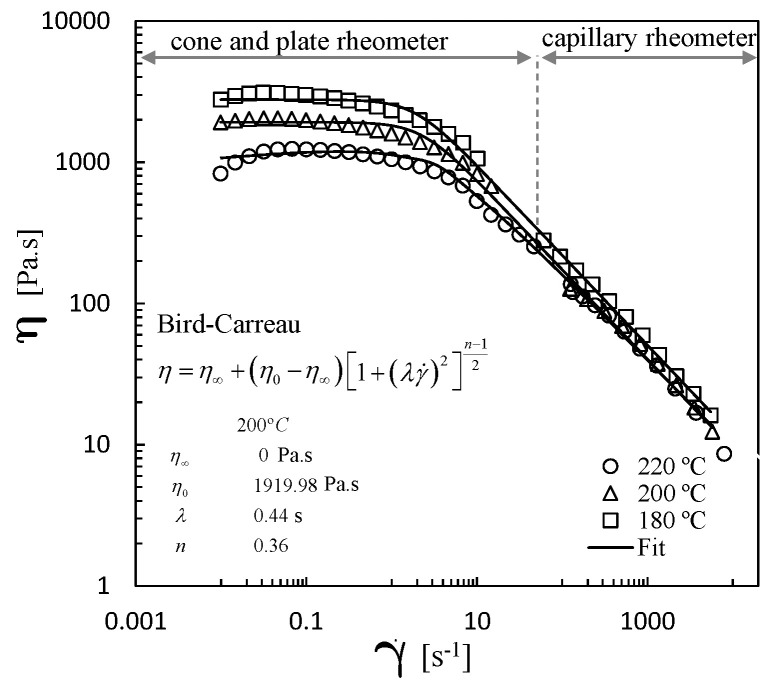
Shear viscosity curves for PP at three different temperatures. Fit with the Bird–Carreau model.

The shear viscosity curves for the three temperatures are displayed in Figure 4, which contains the two sets of data. The fit to the experimental data using the Bird–Carreau model is also shown. From the latter, the zero shear rate plateau ($\eta_{0}$) was obtained, so that the shift factors ($a_{T}$) needed for the construction of master curves could be calculated by

(7) $$a_{T}\left(T\right)=\frac{\eta_{0}\left(T\right)}{\eta_{0}\left(T_{ref}\right)}$$

for each temperature *T*. $T_{ref}$ is the reference temperature of 200 ${}^{\circ }$C.

**Figure 5:**
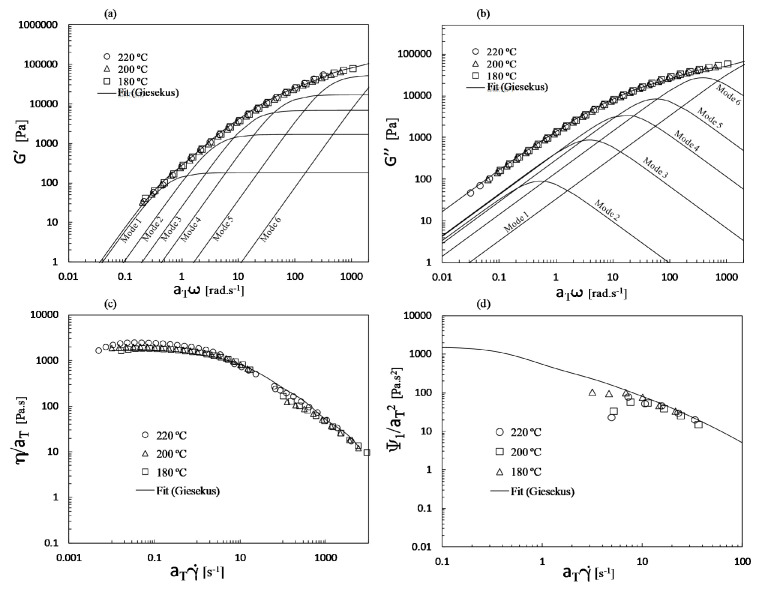
Master curves for PP of the: (**a**) storage modulus; (**b**) loss modulus; (**c**) shear viscosity; (**d**) first normal stress coefficient. The solid line represents the fit obtained with the six-mode Giesekus model.

Figure 5 presents the master curves for the storage modulus, loss modulus, viscosity and first normal stress coefficient.

The solid line represents the fit obtained with the six-mode Giesekus model (see parameters in Table 1). These master curves were also used for the nanocomposite, as the characterization of the rheological behavior for distinct dispersion levels would be quite difficult to perform experimentally. Indeed, this would entail preparing nanocomposites with different, but controlled and homogeneous dispersion levels (while keeping constant the filler concentration). While that would already be a challenge, we would then have to face the additional but well-known re-agglomeration of the filler taking place as we heat the material in the rheometer. This remains an open problem. Even if such a characterization was done, the usefulness of the data would be limited, since in practice nanocomposites prepared by melt mixing do not have a homogeneous state of dispersion, but exhibit a morphology comprising aggregates of different sizes and individual particles.

Moreover, it has been shown that the addition of 2 wt.% of GnP should not significantly influence the rheological response [36].

## 5. Results and Discussion

The characteristics of the melt flow through the mixing device are discussed first, in order to describe the performance of the prototype mixer in terms of velocity profile, streamlines, flow type and normal and shear stress fields. First, the numerical results obtained for a 1:4 expansion followed by a 4:1 contraction are discussed, as the latter is a well-known benchmark geometry in computational fluid dynamics. Then, the flow behavior in the 1:8 expansions and 8:1 contractions that were used experimentally to manufacture the PP/GnP nanocomposite are studied. Finally, experimental data concerning the evolution of the dispersion of the GnP agglomerates along the mixer are combined with the computed shear and normal stresses in order to identify mutual correlations.

### 5.1. The 1:4 Expansion and 4:1 Contraction

Both inelastic and viscoelastic fluids were considered. The simulations with the Bird–Carreau model were performed using the fit of the shear viscosity curve shown in Figure 4, whereas the viscoelastic simulations were carried out with the parameters shown in Table 1.

**Figure 6:**
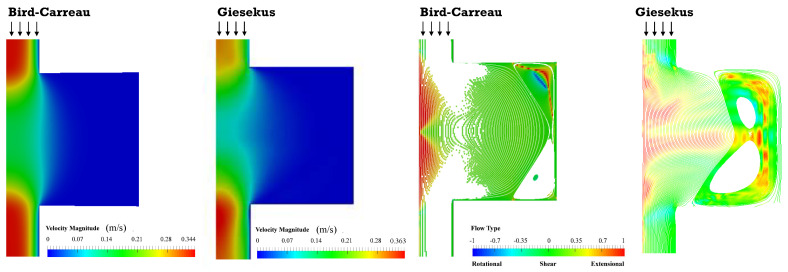
Velocity field, streamlines and flow type parameter obtained for the Bird–Carreau and Giesekus simulations (1:4 expansion/4:1 contraction). In certain regions the number of streamlines is higher, and the white color is more pronounced. Shear flow is predominant in these regions, and therefore, the information on the flow type is not compromised.

Figure 6 depicts the velocity field, the streamlines and the flow type for both inelastic and viscoelastic fluids, for a 1:4 expansion followed by a 4:1 contraction (only half of the channel cross-sections are represented, the flow progressing vertically downwards). As expected, in the smaller channel the fluid velocity is high; the latter reduces in the larger channel due to mass conservation, and makes room for the formation of vortexes. The vortex is bigger and radially asymmetric for viscoelastic fluids, whereas symmetry is preserved for the Bird–Carreau model. Additionally, higher maximum velocities are predicted for the viscoelastic fluid because the velocity profile for the inelastic fluid is closer to plug flow. The flow type results are similar for the two fluids, but the extensional flow distribution is more elaborate for the viscoelastic fluid due to the more complex flow recirculation. It should be remarked that the flow type parameter gives a measure of the intensity of the flow type. When we see a strong elongational flow region, it means that the elongation in that region is stronger than shear (or rotation).

Areas of extensional flow should help with promoting the rupture of solid agglomerates, i.e., dispersive mixing. In turn, the regions of shear flow are responsible for the spatial distribution of the particles (distributive mixing). Anyway, the flow type results should be analyzed cautiously since the magnitude of the velocity should also be taken into account. For example, extensional flow develops in the vortex region, but the local fluid velocity is very low, so the overall contribution to mixing should be negligible.

**Figure 7:**
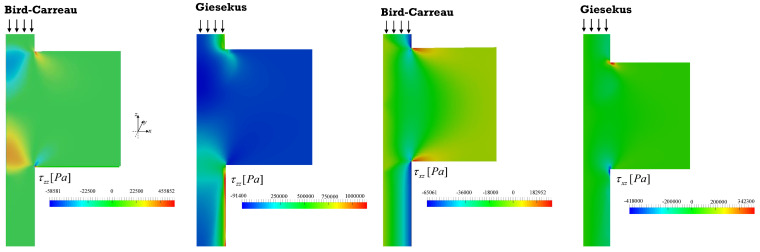
Shear and normal stress fields obtained for the Bird–Carreau and Giesekus simulations (1:4 expansion/4:1 contraction).

**Figure 8:**
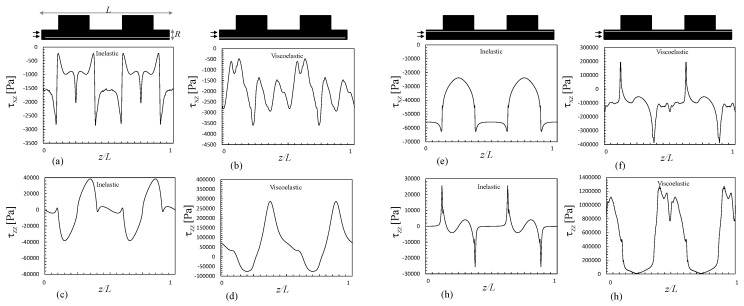
Shear and normal stresses along a mixer consisting of various 1:4 expansions and 4:1 contractions, for the Bird–Carreau (inelastic) and Giesekus (viscoelastic) fluids, at the center x/R≈0 (**a**–**d**) and near the wall (**e**–**h**).

Figure 7 presents the distribution of the hydrodynamic stresses along the same geometry (shear, $\tau_{xz}$, and normal, $\tau_{zz}$, stress fields) for the inelastic and viscoelastic simulations. The fluid suffers severe shear, compression and extension in the z direction. Compression develops near the center when exiting from the smaller to the larger chamber, simultaneously with shear near the wall. Stretching takes place near the center when the fluid exists the chamber and enters into the smaller channel. The magnitudes of the stresses are always higher for the viscoelastic fluid. Stresses require time to relax; thus, before full relaxation is attained (which is assumed to happen instantly for the Bird–Carreau fluid), the fluid is stretched again upon entering the next small channel downstream. These data are detailed in Figure 8, which shows the fluid stresses at the center and near to the channel wall along a sequence of expansions and contractions. The level of the normal stresses can be six times higher than that of the shear stresses near the wall (Figure 8e–h), and up to 200 times higher near the center (Figure 8a–h). Obviously, the shear stress is expected to be zero at the center of the channel. As discussed above, viscoelasticy is associated with higher stresses due to the existence of a relaxation time, as reported also for the extensional flow mixer [13].

The kinematics predicted for this particular flow are similar to the results available in the literature [37,38,39], thereby validating the numerical procedure. The specificity and complexity of this problem makes difficult a straightforward quantitative comparison.

### 5.2. The 1:8 Expansion and 8:1 Contraction

**Figure 9:**
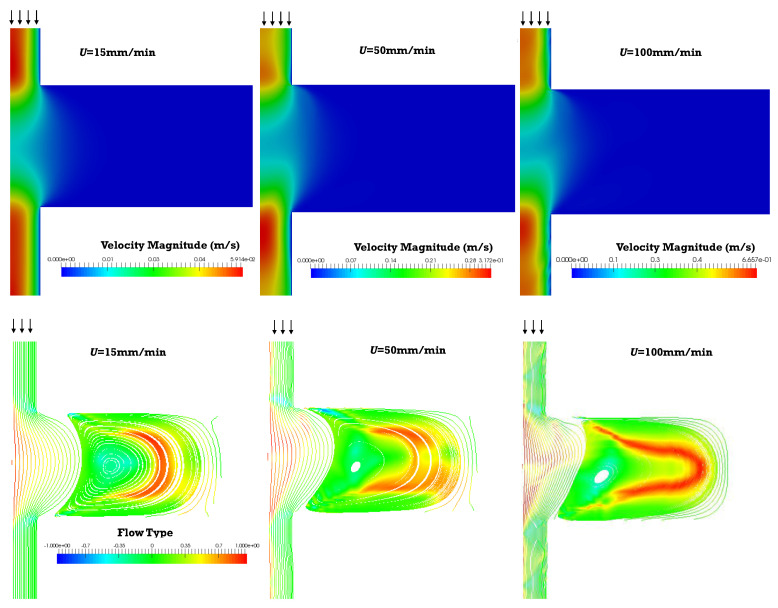
Velocity field (**top**), streamlines and flow type parameter (**bottom**) obtained for the Giesekus simulations, for piston velocities of 15, 50 and 100 m/min (1:8 expansion/8:1 contraction).

**Figure 10:**
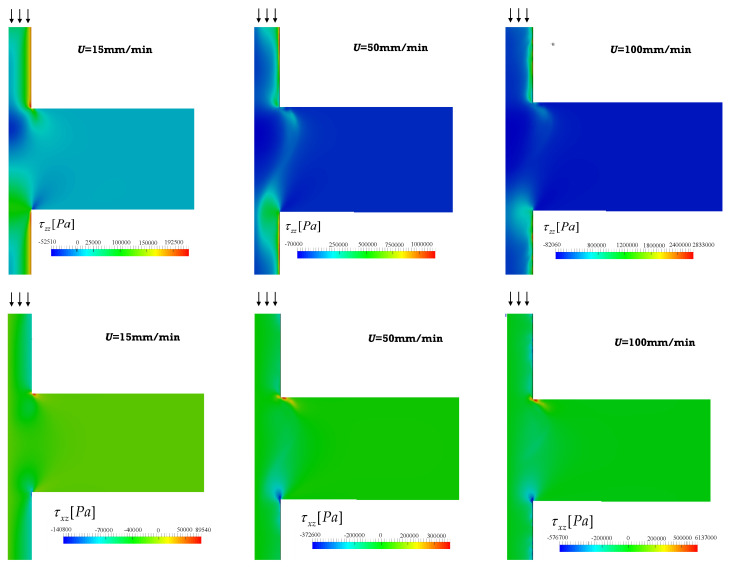
Shear (**top**) and normal stress fields (**bottom**) obtained for the Giesekus simulations, for piston velocities of 15, 50 and 100 m/min (1:8 expansion/8:1 contraction).

The velocity field, flow type, streamlines and shear and normal stresses obtained for the 1:8 expansion and 8:1 contraction, for the piston velocities of 15, 50, and 100 mm/min, are displayed in Figure 9 and Figure 10.

The velocity and stress fields (Figure 9 and Figure 10) are qualitatively similar to those predicted for the 1:4 and 4:1 geometry. Although the majority of shear flow develops in this geometry, the stresses are higher, especially in regions near to channel cross-section changes. Simultaneously, for the same operating conditions, the residence times are also higher, since the diameter of the larger channel is 8 mm instead of 4 mm. The magnitude of the vortexes is also greater and should have a contribution to the distributive mixing.

**Figure 11:**
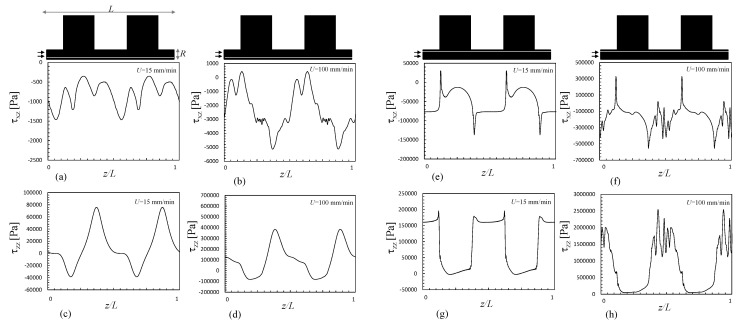
Shear and normal stresses along a mixer consisting of various 1:8 expansions and 8:1 contractions, for a Giesekus viscoelastic fluid, at the center (x/R = 0) (**a**–**d**) and near the wall (x/R = 1) (**e**–**h**). (**a**,**c**,**e**,**f**) piston speed of 15 mm/min; (**b**,**d**,**f**,**h**) piston speed of 100 mm/min.

The shear and normal stresses at the center and near to the channel wall, along a series of expansions and contractions, are shown in Figure 11, for the lowest and highest velocities considered in this work (15 and 100 m/min), assuming a viscoelastic behavior. The global trend is comparable to that of the 1:4/4:1 system (Figure 8), but the usage of high piston speeds can induce much higher stresses. When the piston speed increases from 15 to 100 mm/min, the maximum wall shear stress raises 60% and the maximum normal stress rises 1100%. The increases of the mean shear and normal stresses are 250% and 650%, respectively. These values demonstrate the strong contribution of the singularities at the corners of the channel.

### 5.3. Numerical vs. Experimental Results

As discussed in the introduction, it has been postulated that the solid agglomerates suspended in a liquid will break when the hydrodynamic stresses become significantly higher than their cohesive strength, but there is also a finite probability associated with break-up that is proportional to the residence time and agglomerate surface area [9]. In the present work, the aim was to investigate the trade-off between the experimentally observed evolution of dispersion along a prototype mixer (represented in Figure 3) inducing extensional and shear flows, with the corresponding hydrodynamic stresses adjusted to the time during at which they acted. In order to perform this, the average cumulative residence time along the length of the mixer was measured numerically, considering the predicted velocity fields. Then, at each selected axial location in the mixer, the combined effect of stress and time was estimated from their product. Since both the shear and normal stresses oscillate cyclically along the mixer (see Figure 11), average values were assumed.

**Figure 12:**
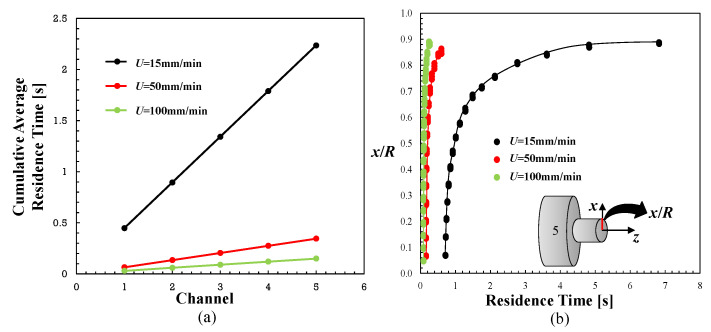
Residence time in the prototype mixer. (**a**) Average cumulative residence along the lengths of the various converging/diverging channels; (**b**) effect of the piston speed on the radial residence time distribution at the outlet of the mixer.

Figure 12a represents the progression of the cumulative average residence time along the length of the mixer. As expected, the residence time increases linearly as the flow advances through the various pairs of larger/smaller channels, but the local residence time in each larger channel is obviously higher than that in the smaller channel (the length of the channels being identical). Figure 12b illustrates the actual radial difference in the local residence times at the mixer outlet. The calculations were performed following the individual streamlines with their respective velocities. As expected, fluid elements at the center of the channel flow more rapidly and are therefore subjected to much lower residence times in the mixer than those nearer to the walls. Thus, the assumption of an average residence time at each cross-section entails some inaccuracy, especially towards the channel edges.

**Figure 13:**
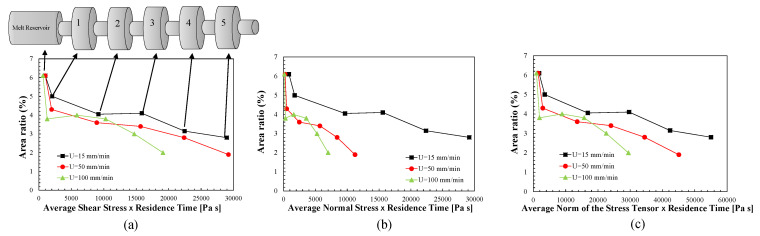
Trade-off between the experimental area ratio (a dispersive mixing index) for a PP/GnP nanocomposite and (**a**) the average absolute value of shear stresses multiplied by the respective residence time; (**b**) the average absolute values of the normal stresses multiplied by the respective residence time; (**c**) the average Frobenius norm of the stress tensor multiplied by the respective residence time. Piston speeds of 15, 50 and 100 mm/min. The mixer consists of a melt reservoir followed by 5 successive pairs of small and larger circular channels.

The trade-off between the local degree of dispersion of the PP/GnP nanocomposite, measured as area ratio, and the absolute value of the hydrodynamic stress multiplied by the local residence time is revealed in Figure 13. The figure shows the correlations obtained for the average shear stresses, the average normal stresses and the average norm of the stress tensor. The latter was calculated as the average Frobenius norm of the stress tensor, given by:

(8) $${\left||\tau |\right|}_{F}={\left(\sum_{i=1}^{3}\sum_{j=1}^{3}{\left|\tau_{ij}\right|}^{2}\right)}^{1/2}.$$

The actual average stress values for the three piston speeds are tabulated in Table 2. A clear quasi-linear relationship between the experimental and the computational data is shown. This confirms that the dispersion of solid agglomerates in a molten viscoelastic matrix requires not only sufficiently high hydrodynamic stresses, but also that these act during sufficient time. Here, the simple multiplicative effect of stress and time was assumed, but other associations could be possible—e.g., a Monte Carlo method of cluster break-up [21]. The contributions of the shear and normal stresses to dispersion seem to be different. The slope of the trends (except for the lowest piston speed) is higher and the values of the stresses are lower in the case of the normal stresses, which seems to confirm the efficacy of extensional flow for dispersion.

Zloczower and Feke [10] showed that the critical stress for dispersion of a regular, body centered cubic, 259-cluster agglomerate depended significantly on cluster size, increasing from a few Pa to over 100 kPa as the cluster size decreases. Santos et al. [40] studied the dispersion of three GnP grades with distinct flake size in the same PP melt and prototype mixer used here. They reported that the dispersion rate along the mixer was lower for the smaller flakes with higher bulk density, which is in accordance with the theoretical considerations.

Since dispersion occurs even for the lowest piston speed, the cohesive strength of the grade of nanoplates used in the present work seems to stand in the kPa scale. As the residence time in the mixer is quite low, it seems reasonable to hypothesize that dispersion progressed through the rupture route. In this case, Scurati et al. [9] reported that for silica particles, rupture occurred when the stresses were at least five times higher than the values of the cohesive stresses, but higher values could be possible for other materials [10].

On the other hand, the stresses will probably range between the average values and the maximum that is reached instantaneously. The latter can be extracted from the graphs for the lowest piston speed in Figure 11. Thus, it would appear that the cohesive strength of the GnP agglomerates studied is somewhere in the range 5–50 kPa.

## 6. Conclusions

This work investigated the trade-off between the evolution of dispersion (measured as area ratio) of PP/GnP nanocomposites along the length of a mixer, inducing a combination of shear and elongational flows, and the corresponding hydrodynamic stresses, weighted by the time during which they were applied. Numerical simulations using the opensource OpenFOAM software of the flow of a viscoelastic melt (following the Giesekus model) in the mixer accessed the velocity, streamlines, flow type and shear and normal stress fields for the polymer and experimental conditions utilized (i.e., the GnP nanoplates were assumed as massless). A strong quasi-linear relationship between the evolution of dispersion measured experimentally and the computational data was obtained. These results confirmed the hypotheses previously put forward by various authors stating that the dispersion of solid agglomerates requires not only sufficiently high hydrodynamic stresses, but also that these act during sufficient time. From considerations based on the theoretical approach proposed by Scurati et al. [9], the cohesive strength of the GnP agglomerates studied was estimated to be in the range 5–50 kPa.

Finally, we may conclude that this work can be used as a guideline to obtain a good rheological fit to viscoelastic polymer melts, establish a relationship between theory and experimental data from the mixing of GnP agglomerates, estimate cohesive strengths and further understand the complex phenomena of dispersion of solid agglomerates.

## Figures and Tables

**Table 1:** Parameters of the six-mode Giesekus model.

Mode	λ [s]	η [Pa.s]	α
1	0.000230	33.4	0.98
2	0.002606	143.3	0.99
3	0.017290	292.4	0.99
4	0.060507	419.6	0.99
5	0.256092	443.4	0.99
6	1.866503	339.2	0.99

**Table 2:** Average shear and normal stresses and average ${\left||\tau |\right|}_{F}$, for different locations along the channel and three different piston velocities.

Piston Velocity	Channel	Av. Shear Stress [kPa]	Av. Normal Stress [kPa]	Av. ${\left||\tau |\right|}_{F}$ [kPa]
	1	45.1	38.1	76.7
	2	36.6	38.4	66.9
15 mm/min	3	34.6	34.0	64.7
	4	33.8	33.7	63.8
	5	33.3	33.5	63.3
	1	21.7	53.4	319.2
	2	19.5	54.0	296.4
50 mm/min	3	19.0	69.1	290.9
	4	18.8	70.5	288.9
	5	18.7	71.9	288.4
	1	304.7	68.7	445.4
	2	268.8	73.5	407.2
100 mm/min	3	260.6	90.2	398.2
	4	257.4	92.0	394.7
	5	252.5	92.3	388.6
